# Mycobacteriosis in Various Pet and Wild Birds from Germany: Pathological Findings, Coinfections, and Characterization of Causative Mycobacteria

**DOI:** 10.1128/spectrum.00452-22

**Published:** 2022-07-19

**Authors:** Volker Schmidt, Heike Köhler, Kristin Heenemann, Petra Möbius

**Affiliations:** a Clinic for Birds and Reptiles, University of Leipzig, Leipzig, Germany; b Institute of Molecular Pathogenesis, Friedrich-Loeffler-Institute (Federal Research Institute for Animal Health), Jena, Germany; c Institute of Virology, University of Leipzig, Leipzig, Germany; Institut National de Santé Publique du Québec

**Keywords:** *Mycobacterium avium*, *Mycobacterium genavense*, Passeriformes, Psittaciformes, birds, coinfections, genotyping, husbandry, mycobacterioses, pathology

## Abstract

A total of 50 birds diagnosed with mycobacteriosis were examined for pathomorphological lesions, coinfections, and causative agents. Mycobacterial species were identified and isolates differentiated using multilocus sequence typing (MLST) and mycobacterial interspersed repetitive-unit variable-number of tandem-repeat (MIRU-VNTR) analysis. Possible associations between mycobacterial species, pathomorphological findings, coinfections, bird orders, and husbandry conditions were evaluated statistically. Mycobacteria were isolated from 34 birds (13 of 22 Psittaciformes, 12 of 18 Passeriformes, five of six Columbiformes, and four other orders) belonging to 26 species in total. Mycobacterium genavense (Mg) was cultured from 15 birds, Mycobacterium avium subsp. *avium* (Maa) from 20 birds, and Mycobacterium avium subsp. *hominissuis* (Mah) from three birds; hence, four birds had mixed infections. About equal numbers of psittacines and passerines were infected with Ma and Mg. The genetic diversity differed; Mg isolates belonged to one MLST type, Maa to six, and Mah to three combined genotypes. Several coinfections were detected; viruses and/or endoparasites affected 44%, fungi 38%, and bacteria 29% of the birds. Pathological findings and mycobacteriosis-affected organs were independent of coinfections. Overall, gross pathological findings were more often seen in mycobacteriosis caused by Ma (95%) compared with Mg (66%). Organ distribution of mycobacteriosis was independent of the mycobacterial species. Pathomorphological changes were seen in the small intestine of 71% and the lung of 65% of the birds, suggesting oral or pulmonal ingestion of mycobacteria. There were no associations between mycobacterial species and bird orders or bird husbandry conditions. Not only Mg, but also Maa and Mah, were clearly identified as primary cause of mycobacteriosis in pet birds.

**IMPORTANCE** In this study, the causative agents and confounding factors of mycobacteriosis in a set of pet and some wild birds from Germany were examined. Not only Mycobacterium genavense, but also M. avium subsp. *avium* and M. avium subsp. *hominissuis*, contributed to mycobacteriosis in these birds. Various coinfections did not affect the manifestation of mycobacteriosis. Due to different gross necropsy findings, however, a different pathogenicity of the two species was assumed. New strains of M. avium subsp. *hominissuis* originating from birds were identified and characterized, which is important for epidemiological studies and for understanding the zoonotic role of this pathogen, as the subsp. *hominissuis* represents an increasing public health concern. The study provides some evidence of correlation between M. avium subsp. *avium* genotypes and virulence which will have to be confirmed by broader studies.

## INTRODUCTION

Mycobacteriosis of birds is mainly caused by Mycobacterium avium or Mycobacterium genavense and is observed worldwide (1, 2). Usually, the species M. avium is subdivided into the subspecies *avium*, *silvaticum*, *hominissuis*, and *paratuberculosis*, although more recent genomic analyses classify these as synonymous taxons (3). M. avium subsp. *avium* and subsp. *silvaticum* are isolated mainly from birds and rarely from mammals and humans (4–8). In contrast, M. avium subsp. *hominissuis*, which is rarely described in birds (4–7, 9–11), is an important opportunistic nontuberculous mycobacterium for humans, pigs, and other mammals, and M. avium subsp. *paratuberculosis* is an important ruminant pathogen. Furthermore, M. genavense has also been recognized as an opportunistic pathogen for humans (12).

The main source of infection for birds, mammals, and humans is usually the environment, including soil, dust, water, and biofilms contaminated with these ubiquitous pathogens, which are highly resistant to environmental challenges (9, 13–15). Fecal excretion of mycobacteria by infected birds leads to contamination of the environment, thus, infection of birds usually occurs through oral ingestion (2, 13, 16). In addition, there is rare evidence of pulmonary infection by inhalation of aerosolized mycobacteria, a possible route of bird-to-bird transmission (9, 13, 14, 17, 18). After oral ingestion of mycobacteria, the intestines become infected leading to a spread to visceral organs and bone marrow, causing granulomatous inflammation (2). In most cases, the infection is associated with predisposing factors of the host, including immunosuppression (17, 19–21). Birds infected with polyomavirus or circovirus may be more susceptible to infection with M. genavense (20, 21).

Several molecular approaches have been used to differentiate the isolates and study the molecular epidemiology of M. avium subsp. *avium* and subsp. *hominissuis*. (6, 7, 9, 11, 14, 16, 22–26). Genetic analysis of various M. avium isolates from humans, mammals, and birds showed a higher diversity of subsp. *hominissuis* than subsp. *avium*, without strict restriction of individual genotypes to geographic regions or host species (6, 7, 9, 11, 14, 16, 22–25). A few molecular studies on M. avium subsp. *avium* isolates and only one on M. genavense isolates, originating from birds that were epidemiologically linked, have been conducted (9, 11, 14, 16, 23). The isolates of these studies were obtained from diseased birds from two zoological gardens, one flock of hens and four flocks of pheasants located in the United States and in the Czech Republic. Disease outbreaks seem to be more often associated with particular M. avium subsp. *avium* genotypes, which supports the likelihood of genotypes with higher virulence (9, 14, 19). Furthermore, mycobacteriosis caused by multigenotype mixed infections was also reported for M. avium subsp. *avium* and for M. genavense (9, 11, 16).

Because the relevance of coinfections in birds suffering from natural M. avium subsp. *avium* infection has not been studied, the purpose of the current study was to assess and compare natural mycobacteriosis cases in pet and some wild birds from Germany caused by M. avium and M. genavense with regard to gross necropsy findings, tissue predilection, coinfections and comorbidities, host bird orders and bird species, and bird husbandry conditions. Furthermore, because M. avium subsp. *hominissuis* is rarely described in birds, the occurrence of this subspecies was examined. In addition, the study aimed to elucidate the genetic diversity of M. avium and M. genavense isolates from German pet birds, and to investigate a possible association between individual genotypes and severity of disease, host bird order, and other factors.

## RESULTS

### Mycobacterial isolates description.

Thirty-eight mycobacterial isolates were obtained from 34 out of the 50 individual birds with mycobacteriosis (Table 1). The isolates were specified as M. genavense in 15 birds of 11 different species belonging to the orders Psittaciformes (seven birds, four species), Passeriformes (seven birds, six species), and Falconiformes (*n* = 1). Isolates of M. avium were obtained from a total of 22 birds of 18 different species, including seven orders, Psittaciformes (six birds, five species), Passeriformes (seven birds, five species), Columbiformes (five birds, four species), Musophagiformes (one bird), Galliformes (one bird), Falconiformes (one bird), and Pelicaniformes (one bird). Both mycobacterial species were obtained from mixed infections detected in two estrildid species (Passeriformes) and a wild juvenile common kestrel (Falconiformes), all included in the listing above (Table 1). Mycobacterial species was independent from gender or husbandry condition of the birds. Remarkably, all five wild birds were infected with M. avium subsp. *avium*; in addition, one of these birds was infected with subsp. *hominissuis* and one was also infected with M. genavense. Apart from this, no other mycobacterial species could be isolated from any of the birds.

**TABLE 1:** List of birds belonging to seven orders and 35 species suffering from mycobacterioses, isolates studied, and mycobacteria species identified

Bird no.^*l*^	Bird order and species	Age	Gender	Origin	Bird husbandry	Coinfections and comorbidities	Isolate no.	Organ	Mycobacteria identified
	Psittaciformes (*n* = 22; 44%)								
1	Budgerigar (Melopsittacus undulatus)	Adult	Female	NW^*e*^	Flock, in- and outdoor	Avian polyomavirus, Macrohabdus ornithogaster, ovarian cysts	17MA0974	Liver	-^*p*^
2	Budgerigar	Adult	Male	SN^*h*^	Flock, household	NAF^*k*^	17MA0785	Small intestine	-
3	Budgerigar	Adult	Male	ST^*i*^	Flock, household	NAF	17MA0787	Spleen	Mg^*m*^
4	Budgerigar	Adult	Female	ST	Flock, in- and outdoor	Avian polyomavirus, Escherichia coli, Macrorhabdus ornithogaster, ovarian cysts, abdominal hernia	18MA1542	Small intestine	Mg
5	Budgerigar	Adult	Female	ST	Flock, in- and outdoor	Avian polyomavirus, Enterobacter cloacae, Macrorhabdus ornithogaster	18MA1549	Liver	Mg
							18MA1550	Small intestine	Mg
6	Budgerigar	Adult	Male	BE^*a*^	Flock, in- and outdoor	Avian polyomavirus, Macrorhabdus ornithogaster, feather mites, feather lices	18MA0517	Liver	Mg
7^*a*^	Red-crowned parakeet (Cyanoramphus novae\ill\elandiae)	Adult	Male	RP^*g*^	Flock, in- and outdoor	NAF	17MA0765	Liver	-
8^*a*^	Red-crowned parakeet	Adult	Female	RP	Flock, in- and outdoor	*Procnemidocoptes janssensi*	17MA0766	Spleen	-
9^*c*^	Red-crowned parakeet	Adult	Female	NW	Single bird, household	NAF	17MA0777	Small intestine	Mah^*o*^
10^*c*^	Red-crowned parakeet	Adult	Male	NW	Single bird, household	*Macrorhabdus ornithogaster*, *Procnemidocoptes janssensi,* amyloidosis	17MA0781	Small intestine	Maa^*n*^
11	Yellow-crowned parakeet (Cyanoramphus auriceps)	Adult	Male	RP	Single bird, household	Macrorhabdus ornithogaster	17MA0768	Lung	-
12	Fische's lovebird (Agapornis fischeri)	Adult	Female	SN	Flock, household	NAF	17MA0763	Spleen	-
13^*f*^	Fischer's lovebird	Adult	Female	SN	Single bird, household	fractured ulna	17MA0771	Spleen	Maa
14^*f*^	Yellow-collared lovebird (Agapornis personatus)	Adult	Female	SN	Flock, household	Polyomavirus, Macrorhabdus ornithogaster	17MA0772	Spleen	Maa
15	Rosy-faced lovebird (Agapornis roseicollis)	Adult	Male	SN	Paired, household	Avian polyomavirus	18MA1543	Liver	Maa
16	Pacific parrotlet (Forpus coelestis)	Adult	Female	NW	Flock, household	Escherichia coli	17MA0775	Spleen	-
17^*b*^	Blue-winged parrotlet (Forpus xanthopterygius)	Adult	Male	NW	Flock, household	Polyomavirus	17MA0778	Liver	-
18^*b*^	Blue-winged parrotlet	Adult	Female	NW	Flock, household	Polyomavirus	17MA0971	Small intestine	Maa
19	Australian king parrot (Alisterus scapularis)	Adult	Male	SN	Single bird, household	Klebsiella oxytoxca	17MA0758	Small intestine	Mg
20	Scaly-headed parrot (Pionus maximiliani)	Adult	Male	SN	Paired, household	NAF	17MA0963	Small intestine	Mg
21	Blue-bellied parrot (Triclaria malachitacea)	Adult	Female	RP	Paired, in- and outdoor	Aspergillus fumigatus	17MA0964	Lung	-
22	Swift parrot (Lathamus discolor)	Adult	Female	SA^*g*^	Flock, in- and outdoor	Clostridium perfringens, Candida albicans, Macrorhabdus ornithogaster, ascarid nematodes	17MA0973	Spleen	Mg
	Passeriformes (*n* = 18; 36%)								
23	European goldfinch (Carduelis carduelis)	Adult	Male	BY^*c*^	Flock, in- and outdoor	Circovirus, Macrorhabdus ornithogaster, visceral coccides, cestodes,	17MA0774	Liver	Maa
24	European goldfinch	Adult	Male	NW	Flock, in- and outdoor	Polyomavirus, circovirus, Macrorhabdus ornithogaster	19MA0130	Granulom serosa	Maa
25	Canary (Serinus canaria forma domestica)	Adult	Male	SN	Flock, in- and outdoor	Canary bornavirus 1	17MA0759	Liver	-
26	European serin (Serinus serinus)	Adult	Female	SN	Flock, in- and outdoor	Escherichia coli, Macrorhabdus ornithogaster, visceral coccids	18MA1547	Spleen	Mg
							18MA1548	Small intestine	Mg
27	Eurasian bullfinch (Pyrrhula pyrrhula)	Adult	Male	BW^*b*^	Flock, in- and outdoor	Enterobacter cloacae, Aspergillus fumifatus	17MA0762	Lung	-
28	European starling (Sturmus vulgaris)	Adult	Female	ST	Wild bird, outdoor	Polyomavirus, visceral coccids, Giardia duodenalis, cestodes, feather lice, fractured beak	18MA1544	Liver	Maa
							18MA1545	Joint	Maa
							18MA1546	Bone	Maa
29	European starling	Juvenile	Female	SN	Wild bird, outdoor	Usutu virus, Escherichia coli, Candida albicans, enteral coccids, cestodes, fractured humerus	19MA0132	Liver	Maa
30^*g*^	European robin (Erithacus rubecula)	Adult	Male	SN	Wild bird, outdoor	Finch polyomavirus, visceral coccids, feather mites, fractured ulna	17MA0761	Liver	Mah
31^*g*^	Green-backed twinspot (Mandingoa nitidula)	Adult	Male	SN	Flock, household	Polyomavirus, cestodes	19MA0129	Granulom serosa	Maa/Mg
32^*c*^	Green-backed twinspot	Adult	Female	SN	Flock, household	Polyomavirus, cestodes	19MA0131	Liver	Mg
33	Pin-tailed parrot finch (Erythrura prasina)	Adult	Male	SN	Flock, household	cryptosporida, cestodes	17MA0760	Spleen	-
34	African firefinch (Lagonosticta rubricata)	Adult	Female	BY	Flock, household	visceral coccids, cestodes, amyloidosis	17MA0782	Small intestine	Maa/Mg
35^*c*^	Black-rumped waxbill (Estrilda troglodytes)	Adult	Female	SN	Flock, household	Candida albicans, cryptosporida, visceral coccids, cestodes, amyloidosis	17MA0764	Lung	Mg
36	Gouldian finch (Chloebia gouldiae)	Adult	Female	SN	Single bird, household	Polyomavirus	17MA0779	Liver	Mg
37	Green-headed tanager (Tangara seledon)	Adult	Female	TH^*j*^	Flock, household	Escherichia coli, visceral coccids, traumatic brain injury	17MA0767	Liver	Mg
38	Turquoise honeycreeper (Dacnis cayana)	Adult	Female	BE	Flock, household	NAF	18MA0518	Liver	-
							18MA0519	Small intestine	-
39	White-throated magpie-jay (Calocitta formosa)	Adult	Male	ST	Paired, in- and outdoor	Escherichia coli	17MA0773	Small intestine	-
40	Village weaver (Ploceus cucullatus)	Adult	Male	BE	Flock, in- and outdoor	Candida albicans, Aspergillus fumigatus, visceral coccids	17MA0962	Lung	-
	Columbiformes (*n* = 6; 12%)								
41^*d*^	Pink-headed fruit dove (Ptilinopus porphyreus)	Adult	Male	LS^*d*^	Flock, in- and outdoor	Candida albicans	17MA0769	Lung	Maa
42^*d*^	Pink-headed fruit dove	Adult	Male	LS	Flock, in- and outdoor	Escherichia coli	17MA0770	Liver	Maa
43^*d*^	Pink-headed fruit dove	Adult	Female	LS	Flock, in- and outdoor	Escherichia coli, Clostridium baratii	17MA0783	Spleen	-
44	Wompoo fruit dove (Ptilinopus magnificus)	Adult	Male	SN	Flock, in- and outdoor	capillarid nematodes	17MA0776	Spleen	Maa
45	Tambourine dove (Turtur tympanistria)	Adult	Male	BW	Flock, in- and outdoor	NAF	17MA0780	Lung	Maa/Mah
46	Feral pigeon (Coh\ill\mba liviaforma domestica)	Adult	Male	NW	Wild bird, outdoor	Salmonella Typhimurium var. Copenhagen, Ascaridia cohenbae	19MA0128	Lung	Maa
	Musophagiformes (*n* = 1; 2%)								
47	Fischer‘s turaco (Tauraco fischeri)	Adult	Female	SN	Flock, in- and outdoor	enteral coccids	17MA0784	Spleen	Maa
	Galliformes (*n* = 1; 2%)								
48	Chicken (Gallus gallus forma domestica)	Adult	Female	BW	Flock, in- and outdoor	exogenous avianleukosis virus-K	17MA0972	Spleen	Maa
	Falconiformes (*n* = 1; 2%)								
49^*f*^	Common kestrel (Falco tinnunculus)	Juvenile	Male	SN	Wild bird, outdoor	Parahaemoproteus, fractured scapula and dislocation of the shoulder	17MA0788	Liver	Maa/Mg
	Pelicaniformes (*n* = 1; 2%)								
50	Malagasy pond heron (Ardeola idea)	Adult	Female	LS	Flock, in- and outdoor	NAF	17MA0969	Spleen	Maa

a BE, Berlin.

b BW, Baden Würtemberg.

c BY, Bavaria.

d LS, Lower Saxony.

e NW, North Rhine-Westphalia.

f RP, Rhineland-Palatinate.

g SA, Saarland.

h SN, Saxony.

i ST, Saxony-Arhalt.

j TH, Thuringia.

k NAF, no additional findings.

l Identical letters a, b, c, d, birds were from the same flock; (a to d), e, f, g, designate birds with identical regional origin (post code).

m Mg, *M. genavense*.

n Maa, M. avium subsp. *Avium*.

o Mah, M. avium subsp. *Hominissuis*.

p -, no mycobacteria could be identified.

Besides the diagnosis of mycobacteriosis in 50 birds, several coinfections and comorbidities were detected in 41 birds; nine birds were without additional findings (Table 1). Isolates of birds from the same flock belonged to identical mycobacterial species or subspecies (birds no. 32, 35: M. genavense; birds no. 41, 42: M. avium subsp. *avium* with identical genotype), but these birds were affected by different coinfections. In another pair of birds from the same flock, both coinfected with polyomavirus, no mycobacteria could be isolated from one of the two birds. Isolates of birds with identical regional origin represented different subspecies of M. avium as well as variable mycobacterial species (Table 1).

### Pathomorphological characteristics of organ lesions, coinfections, and their association with isolated mycobacterial species.

Overall, gross pathological findings were more often seen in mycobacteriosis caused by M. avium (18 of 19 birds with isolation of M. avium only; 95%) compared with cases with isolation of M. genavense only (eight of 12 birds; 66%). The absence of gross pathological findings was weakly associated in cases with isolation of only M. genavense compared with cases with isolation of only M. avium (*φ* = 0.372, *P* = 0.038).

Main gross necropsy findings were hepatosplenomegaly in 24 of 34 birds (71%), emaciation in 22 of 34 birds (65%), and/or proliferative white to yellow nodular capsulated foci with caseous content in various visceral organs, ranging from 1 mm up to 25 mm in dimension, which were seen in 19 of 34 birds (56%). The distribution of these necropsy findings was independent of the bird orders of the affected birds. Hepatosplenomegaly, as the most common finding, was commonly combined with these proliferative white foci (16 of 24 birds with hepatosplenomegaly, 67%). Hepatosplenomegaly without proliferative foci was seen in a European robin (*Erithacus rubecula*) with isolation of M. avium subsp. *hominissuis*, in one Blue-winged parrotlet (*Forpus xanthopterygius*) with isolation of M. avium subsp. *avium*, in five birds infected with M. genavense, and in one African firefinch (*Lagonostica rubricata*) infected with both mycobacterial species. Neither proliferative foci nor hepatosplenomegaly were seen in one European goldfinch (*Carduelis carduelis*) with isolation of M. avium subsp. *avium*, and in the remaining four birds with isolation of M. genavense (Table 2). Emaciation was seen irrespective of the causative mycobacterial species, hepatosplenomegaly or granulomas. Proliferative white to yellow nodular capsulated foci were seen significantly more often in cases with isolation of M. avium only (16 of 19 birds, 84%; *P* < 0.001; *φ* = 0.743, *P* < 0.001; *OR *= 58.667, *P* = 0.001) compared with cases with isolation of M. genavense only (one of 12 birds, 8%). Additionally, one juvenile wild European starling showed a yellow nodule of 1 cm in diameter filled with caseous material on the right elbow joint. One red-crowned parakeet with isolation of M. avium subsp. *hominissuis* revealed proliferative white foci on the conjunctiva and the cervical air sacs, but not in the visceral organs (Table 2).

**TABLE 2:** Main pathological findings, affected organs, and coinfections in 34 birds infected with M. avium (Ma) and M. genavense (Mg)^*a*^

Main pathological findings, affected organs, and coinfections of 34 birds (absolute and relative no.)	Psittaciformes (*n* = 13)		Passeriformes(*n* = 12)		Others (*n* = 9)	
	Ma (*n* = 6)	Mg (*n* = 7)	Ma (*n* = 7)	Mg (*n* = 7)	Ma (*n* = 9)	Mg (*n* = 1)
Emaciation (22; 65%)	5	6	2^*b*^	6^*b*^	3	0
Granulomatous nodules (19; 56%)	5	1	4^*c*^	1^*c*^	8^*e*^	1^*e*^
Hepatosplenomegaly (24; 71%)	6	2	6^*b*^	6^*b*^	4	0
Amyloidosis (4; 9%)	1	0	1^*d*^	2^*d*^	0	0
Without nodules and hepatosplenomegaly (5; 15%)	0	1	1	3	0	0
Trauma (6; 18%)	1	0	3	1	1^*e*^	1^*e*^
Affected organs						
Various visceral organs (29; 85%)	6	5	7^*b*^	6^*b*^	5^*e*^	1^*e*^
Small intestine (24; 71%)	4	5	6^*b*^	6^*b*^	3^*e*^	1^*e*^
Lung (22; 65%)	5	2	5^*c*^	6^*c*^	4	0
Viruses (15; 44%)						
Polyomavirus (12 of 25 tested birds; 48%)	3	3	4^*c*^	3^*c*^	n.d.	n.d.
Circovirus (1 of 25 tested birds; 4%)	0	0	2	0	n.d.	n.d.
Usutu virus (1 of 12 tested birds; 8%)	n.d.^*f*^	n.d.	1	0	n.d.	n.d.
Exogenous avian leucosis virus-K	n.d.	n.d.	n.d.	n.d.	1	n.d.
(1 of 1 tested bird)						
Bornavirus (0 of 25 tested birds)	0	0	0	0	n.d.	n.d.
Endoparasites (15; 44%)	0	1	6^*b*^	6^*b*^	3^*e*^	1^*e*^
Cestodes (9; 26%)	0	0	5^*b*^	4^*b*^	0	0
Nematodes (3; 9%)	0	1	0	0	2	0
Enteral coccids	0	0	1	1	1	0
Visceral coccids	n.d.	n.d.	4^*d*^	4^*d*^	0	0
*Parahaemoproteus* sp.	0	0	0	0	1^*e*^	1^*e*^
Giardia intestinalis	0	0	1	0	0	0
Ectoparasites (3; 9%)	1	1	1	0	0	0
Feather mites	0	1	0	0	0	0
Feather lice	0	1	1	0	0	0
*Procnemidocoptes janssensi*	1	0	0	0	0	0
Fungi (13; 38%)	2	4	4^*d*^	3^*d*^	1	0
*Macrorhabdus ornithogaster*	2	4	3^*d*^	2^*d*^	0	0
Candida albicans	0	1	1	1	1	0
Bacteria (10; 29%)	0	4	1	3	2	0
Escherichia coli	0	1	1	2	1	0
Klebsiella oxytoca	0	1	0	0	0	0
Enterobacter cloacae	0	1	0	1	0	0
Clostridium perfringens,	0	1	0	0	0	0
Salmonella Typhimurium var. Copenhagen)	0	0	0	0	1	0
Without coinfection (5; 15%)	1	2	0	0	2	0

a The findings are listed by the bird orders Psittaciformes and Passeriformes and other birds belonging to the orders Columbiformes, Musophagiformes, Galliformes, Falconiformes, and Pelicaniformes.

b Infection with both mycobacterial species in two estrildid finches incl. one.

c Green-backed twinspot (*Mandingoa nitidula*) and one.

d African firefinch (*Lagonosticta rubricate*).

e Infection with both mycobacterial species in one wild juvenile common kestrel (*Falco tinnunculus*).

f n.d., not determined.

Histopathology of foci confirmed mycobacterial tubercles in the form of fibrinous granulomas, characterized by necrosis and fibrin in the center, surrounded by multinucleated giant cells, histiocytes, lymphocytes, and heterophils as well as various degrees of fibroplasia. Acid-fast rod-shaped bacteria were detected in the center of the granulomas as well as intracytoplasmic in the multinucleated giant cells. The birds without any proliferative foci showed accumulations of epithelioid cell-like macrophages with acid-fast rod-shaped bacteria in various organs.

The organ distribution of mycobacteriosis in individual birds was independent of the isolated mycobacterial species. Furthermore, a comparison between Passeriformes and Psittaciformes showed no difference in organ distribution (Table 2). Besides liver and spleen, various visceral organs were infected in 29 of 34 (85%) birds. Pathomorphological correlation of mycobacterial infection was seen in the small intestine of 24 of 34 (71%) birds and in the lung of 22 of 34 (65%) birds.

Viral coinfections and/or endoparasitoses (15 of 34 birds each; 44%) were most common, especially with polyomaviruses in passerines and psittacines (six birds each) and visceral coccids and/or cestodes in passerines (seven of 12 passerines each; 58%) (Table 2). These findings were irrespective of the isolated mycobacterial species, pathological findings and other coinfections.

Fungal coinfections (13 of 34 birds; 38%) and bacterial coinfections (10 of 34 birds; 29%) were less common than viral coinfections and/or endoparasites (Table 2). Contrary to fungal coinfections, which were irrespective of the isolated mycobacterial species, bacterial coinfections were more common in birds with M. genavense infection (six of 12 birds with isolation of M. genavense only; 50%; *φ* = 0.367; *P* = 0.041) compared with birds with isolation of M. avium (three of 19 birds with isolation of M. avium only; 16%).

No additional pathogens were detected in a total of five birds (15%) belonging to Psittaciformes, Columbiformes, and Pelicaniformes, while all passerine birds showed coinfections. Pathological findings and organ distribution of mycobacteria were independent of whether or not coinfection was present. Monoinfection only with mycobacteria, with either M. avium (*n* = 3) or M. genavense (*n* = 2), was diagnosed in three of 13 psittacines (23%), in the Tambourine dove (*Turtur tympanistria*), and in the Malagasy pond heron (*Ardeola idea*), (Table 1 and 2). It should be noted here that a single-kept red-crowned parakeet (one of the psittacines) was infected with M. avium subsp. *hominissuis* only and the Tambourine dove was infected with both M. avium subspecies.

Additionally, a traumatic injury was seen in six of 34 birds (18%) including four wild birds. Deposition of amyloid in visceral organs was observed in three birds with various mycobacterial species and coinfections (Table 2).

### M. avium genotypes.

Subspecies identification revealed M. avium subsp. *avium* in 20 birds, including both juvenile wild birds, and M. avium subsp. *hominissuis* in three birds: an adult free-living European robin (bird no. 30), a single red-crowned parakeet (bird no. 9) kept indoors, and a Tambourine dove (bird no. 45) from a flock kept in an in- and outdoor aviary (Table 1 and 3).

**TABLE 3:** Mycobacteria isolates, their origin, and genotypes based on MIRU-VNTR-analysis and MLST

Mycobacteria identified	Isolate no.	Bird no.^*d*^	Bird order	Tissue	MIRU-VNTR INMV profile^*e*^	MLST ST^*f*^
Maa^*a*^	17MA0781	10	Psittaciformes	Small intestine	67	ST40
	17MA0771	13	Psittaciformes	Spleen	67	ST40
	17MA0772	14	Psittaciformes	Spleen	67	ST40
	18MA1543	15	Psittaciformes	Liver	67	ST40
	17MA0971	18	Psittaciformes	Small intestine	100	ST22
	17MA0774	23	Passeriformes	Liver	67	ST40
	19MA0130	24	Passeriformes	Granuloma serosa	100	ST21
	18MA1544	28^w^	Passeriformes	Liver	67	ST21
	18MA1545	28^w^		Joint	67	ST21
	18MA1546	28^w^		Bone	67	ST21
	19MA0132	29^w^	Passeriformes	Liver	90	ST23
	17MA0776	44	Columbiformes	Spleen	67	ST40
	19MA0128	46^w^	Columbiformes	Lung	100	ST21
	17MA0784	47	Musophagiformes	Spleen	67	ST40
	17MA0972	48	Galliformes	Spleen	100	ST22
	17MA0969	50	Pelicaniformes	Spleen	100	ST21
	17MA0769	41	Columbiformes	Lung	100	ST22
	17MA0770	42	Columbiformes	Liver	100	ST22
						
Mah^*b*^	17MA0777	9	Psittaciformes	Small intestine	new1	ST51
	17MA0761	30^w^	Passeriformes	Liver	new2	ST60
						
Maa/Mah	17MA0780	45	Columbiformes	Lung	new3/93	ST40/ST50
						
Maa/Mg	19MA0129	31	Passeriformes	Granuloma serosa	100/-	ST21/no result
	17MA0782	34	Passeriformes	Small intestine	no result	no result/type 1
	17MA0788	49^w^	Falconiformes	Liver	67/-	ST21/type 1
						
Mg^*c*^	17MA0787	3	Psittaciformes	Spleen	-^*g*^	type 1
	18MA1542	4	Psittaciformes	Small intestine	-	type 1
	18MA1549	5	Psittaciformes	Liver	-	type 1
	18MA1550	5		Small intestine	-	type 1
	18MA0517	6	Psittaciformes	Liver	-	type 1
	17MA0758	19	Psittaciformes	Small intestine	-	type 1
	17MA0963	20	Psittaciformes	Small intestine	-	type 1
	17MA0973	22	Psittaciformes	Spleen	-	type 1
	18MA1547	26	Passeriformes	Spleen	-	type 1
	18MA1548	26		Small intestine	-	type 1
	19MA0131	32	Passeriformes	Liver	-	type 1
	17MA0764	35	Passeriformes	Lung	-	type 1
	17MA0779	36	Passeriformes	Liver	-	type 1
	17MA0767	37	Passeriformes	Liver	-	type 1
						

a Maa, M. avium subsp. *avium.*

b Mah, M. avium subsp. *hominissuis*.

c Mg, M. genavense.

d Letter w, designate wild living birds.

e INMV profiles according to classification database.

f MLST, subtype (ST) designation for M. avium isolates according to (25, 36); MLST established for M. genavense (this study) showed only one type.

g -, not determined (because no specific MIRU-VNTR for Mg was available).

The M. avium subsp. *avium* isolates were differentiated into five MIRU-VNTR based INMV profiles (Table 4). The already known profiles INMV67 (*n* = 9) and INMV100 (*n* = 7) represented nearly all subsp. *avium* isolates. INMV90 was determined only once. In addition, there were two so far unknown profiles in isolates with mixed MIRU-VNTR results. One of these isolates (17MA0780) contained both M. avium subspecies, the other isolate (17MA0788) belonged to M. avium subsp. *avium* and M. genavense. The M. avium subsp. *avium* clone of isolate 17MA0788, originating from the juvenile wild kestrel, showed two alleles at MIRU-VNTR locus 32 only and no mixed MLST genotype. This suggests a polymorphic structure at this locus rather than a mix of very similar subsp. *avium* clones.

**TABLE 4:** MIRU-VNTR genotypes shown with individual profiles identified in this study

INMV profile	No. of repeats at MIRU-VNTR loci								Subspecies variant
	292	X3	25	47	3	7	10	32	
67	2	3	1	3	1	1	2	7	Maa^*a*^
90	2	2	1	3	1	1	2	7	Maa
100	2	4	1	3	1	1	2	7	Maa
^*c*^67/New*^1^^*d*^	2	3	1	3	1	1	2	7/8	Maa
New1	2	2	3	2	1	1	2	8	Mah^*b*^
New2	0	4	3	3	1	1	2	8	Mah
^*c*^New3/93*^2^^*e*^	0/2	2	1/2/3	2	1	1	2	8/9	Maa/Mah

a Maa = M. avium subsp. *avium*.

b Mah = M. avium subsp. *hominissuis*.

c mixed genotypes.

d 67/New*^1^, for this mixed genotype at VNTR32 no mixed MLST genotype was found.

e New3/93*^2^, new3 for Maa: 22121128 (suspected), for Mah: 22221129 = INMV93 (suspected), a third genotype with 0 repeats at locus 292 and 3 repeats at locus 25 is suspected.

Using MLST, subsp. *avium* isolates belonged to four sequence types: ST40 (*n* = 8), ST21, ST22 (each *n* = 4), and ST23 (*n* = 1) that were based on sequence variances in three target genes with three (*recF*), two (*lipT*), and two (“*est*”) different sequence alleles (Table 5; Table S3).

**TABLE 5:** M. avium sequence types (ST) and their allelic profiles^*a*^

ST	Allelic profile	Subspecies
ST21	6-7-4-5-3	Maa^*b*^
ST22	6-7-4-5-10	Maa
ST23	7-7-5-5-3	Maa
ST40	8-7-4-5-3	Maa
ST50	5-3-1-1-11^*d*^	Mah^*c*^
ST51	13-10-1-8-1^*d*^	Mah
ST60	5-3-3-1-11^*d*^	Mah

a STs and allelic profiles based on sequence variances at MLST loci *recF*, *gnd1*, *lipT*, *pepB*, and “*est*” according to (25), completed for locus “*est*” in this study; all SNPs shown in Table S3.

b Maa = M. avium subsp. *avium*.

c Mah = M. avium subsp. *hominissuis*.

d Alleles for locus “*est*” new determined in this study.

The two individual M. avium subsp. *hominissuis* strains were characterized by two new MIRU-VNTR profiles (Table 4) never published before, and by two MLST sequence types (ST51 and ST60; Table 5). Here, results at the MIRU-VNTR loci 292, X3, and 47 revealed differently sized alleles, and MLST showed sequence variances at five target genes (*recF*, *gnd1*, *lipT*, *pepB*, and *“est”*) each with two differently distributed sequence alleles (Table S3).

The MIRU-VNTR genotypes of different subspecies within the mixed isolate 17MA0780 could not be clearly identified. It is assumed that M. avium subsp. *hominissuis* (17MA0780a) includes the profile INMV 93, and subsp. *avium* (17MA0780b) includes the new profile 22121128 (new3; Table 4). In addition, a third genotype is suggested. Using MLST, manual analysis of single nucleotide polymorphisms (SNPs) at specific variable positions in the sequence chromatograms of this isolate showed mixed nucleotides resulting in ST50 for M. avium subsp. *hominissuis* in addition to the ST40 for M. avium subsp. *avium* (Table 6).

**TABLE 6:** Combined genotypes of M. avium isolates

Genotype combined	INMV profile	ST	Subsp. variant	Bird strains with respective genotype
1	67	ST21	Maa^*a*^	18MA1544^*c*^, 18MA1545^*c*^, 18MA1546^*c*^, 17MA0788^*d*^
2	67	ST40	Maa	17MA0771, 17MA0772, 17MA0774, 17MA0776
				17MA0781, 17MA0784, 18MA1543
3	90	ST23	Maa	19MA0132
4	100	ST21	Maa	17MA0969, 19MA0128, 19MA0129, 19MA0130
5	100	ST22	Maa	17MA0769, 17MA0770, 17MA0971, 17MA0972
6	new1	ST51	Mah^*b*^	17MA0777
7	new2	ST60	Mah	17MA0761
8	93	ST50	Mah	17MA0780a
9	new3	ST40	Maa	17MA0780b

a Maa, M. avium subsp. *avium*.

b Mah, M. avium subsp. *hominissuis*.

c Isolates originating from different tissues of bird 28 (see Table 3).

d 17MA0788 included two alleles at MIRU-VNTR locus 32 (7 and 8) but no mixed results using MLST.

A combination of MIRU-VNTR and MLST typing resulted in six and three different combined genotypes for M. avium subsp. *avium* and for subsp. *hominissuis*, respectively (Table 6).

Using IS*901*-RFLP with BstEII and PvuII digestion for additional characterization of several strains with identical combined genotypes (*n* = 4), four combined band patterns were detected (Fig. S1 and S2). These four patterns could be assigned to four individual combined genotypes confirming the previous differentiation of isolates using results of MIRU-VNTR analysis and MLST (Table 7).

**TABLE 7:** IS*901*-RFLP patterns detected for several M. avium subsp. *avium* isolates with specific combined genotypes

Combined genotypes	MIRU-VNTR/MLST types	IS*901*-RFLP pattern^*a*^
1	INMV67/ST21	B3-Z2
2	INMV67/ST40	B1-Z1
4	INMV100/ST21	B4-Z3
5	INMV100/ST22	B2-C

a IS*901*-RFLP pattern after digestion with BstEII and PvuII; see Fig. S1 and S2.

All birds with combined genotype 4 (INMV100-ST21; *n* = 4) showed no clinical signs, and all birds with combined genotype 2 (INMV67-ST40; *n* = 7) presented with poor body condition and visceral granulomas.

### Cluster analysis of M. avium isolates.

**FIG 1:**
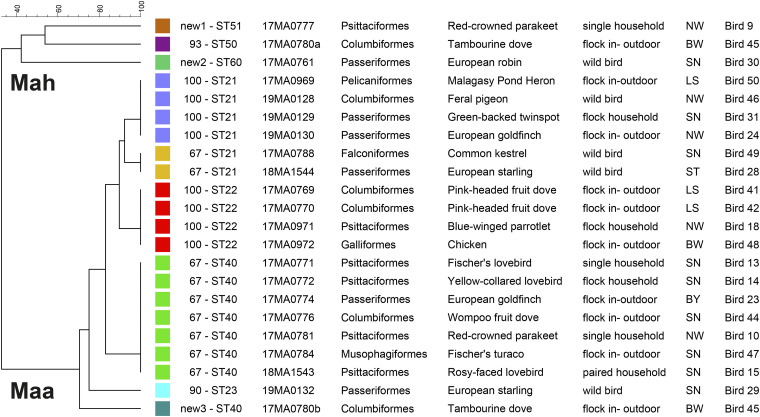
Phylogenetic tree representing genetic similarities of nine combined genotypes of M. avium isolates from 21 birds, their order, species, type of bird husbandry, and regional origin. The dendrogram was generated with unweighted pair group method using arithmetic averages analyses based on MIRU-VNTR and MLST typing. Isolates are differentiated into two distinct groups: M. avium subsp. *hominissuis* (Mah) and M. avium subsp. *avium* (Maa).

**FIG 2:**
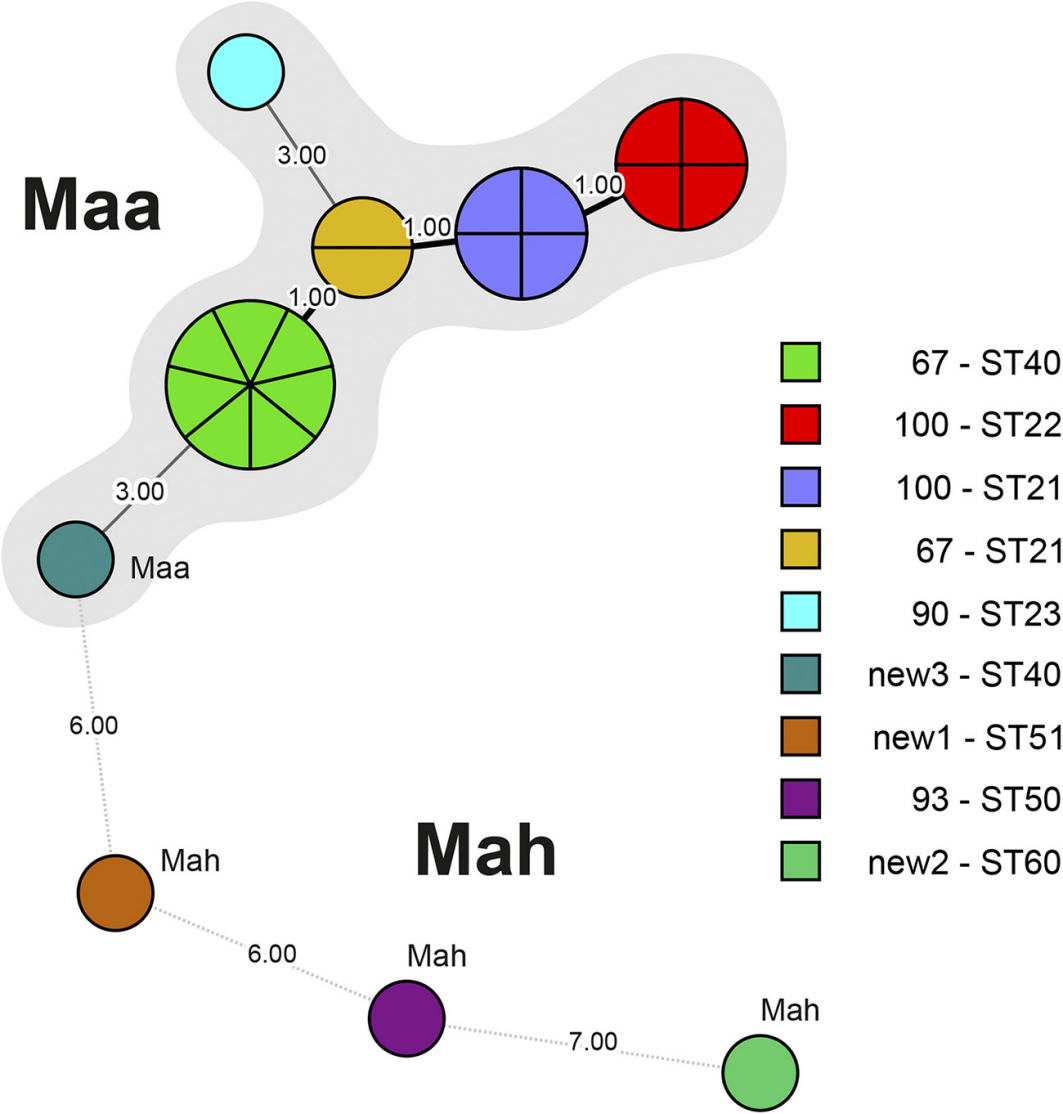
Minimum spanning tree (MST) based on results of combined genotyping (MIRU-VNTR and MLST) of M. avium isolates from 21 birds. Circle sizes are proportional to the number of isolates with identical pattern. Numbers between the circles represent the genetic distances between M. avium subsp. *avium* (Maa) and M. avium subsp. *hominissuis* (Mah) isolates.

The dendrogram (using UPGMA) and the minimum spanning tree, both based on MIRU-VNTR and MLST genotypes, represent the genetic similarities and the relationships among the 21 M. avium bird isolates, including a mixed isolate (Fig. 1 and 2). Mycobacterium avium isolates are clearly separated into two distinct clusters of subsp. *avium* and *hominissuis* and the higher similarity among M. avium subsp. *avium* than among M. avium subsp. *hominissuis* genotypes are highlighted.

### M. genavense genotype.

Mycobacterium genavense isolates (*n* = 17) originating from 15 individual birds also including three mixed isolates with M. avium showed no sequence differences in the seven target genes selected for MLST. Thus, these isolates could not be differentiated by MLST and show only one type (Table 3).

## DISCUSSION

### Mycobacteria in pet and wild living birds.

In this study, pathological findings, coinfections, and comorbidities in pet birds and some wild birds affected by mycobacteriosis were investigated and the causative agents identified and characterized. In contrast to reports that M. avium subsp*. avium* was identified mainly in domestic birds (27) and M. genavense mainly in pet birds (1, 18, 28, 29), it could be clearly shown here that M. avium subsp. *avium* is an important cause for mycobacteriosis in pet birds, in addition to M. genavense. Both species were also detected in the few wild birds studied. Furthermore, birds belonging to Psittaciformes and Passeriformes were equally affected by both species, in contrast to birds belonging to Columbiformes, all infected by M. avium only. Further studies need to clarify whether Columbiformes are more susceptible to M. avium than to M. genavense.

The results of this study confirm a previous report from the San Diego Zoo and its Safari Park where M. genavense and M. avium (including subsp. *avium* and subsp. *hominissuis*) were the most common mycobacterial species in diseased birds of various species (11). In contrast to other studies determining few cases of other mycobacteria (M. intracellulare, M. fortuitum, M. gordonae, and *M. nonchromogenicum*) as cause for mycobacteriosis in pet birds or zoo birds (1, 11), no other mycobacteria were identified in the current study.

### Pathological findings and their possible association with causative mycobacteria.

Hepatosplenomegaly with or without emaciation and/or granulomas was the main pathological finding and was frequently described in cases of mycobacteriosis in birds (30). The data presented here confirmed that gross pathological findings, especially formation of granulomas, were more often seen in mycobacteriosis caused by M. avium subsp. *avium*. Absence of these findings is more often associated with cases caused by M. genavense irrespective of whether psittacines or passerines were infected (2, 18, 30). Portaels et al. (1996) (31) interpreted similar results as a limited pathogenicity of M. genavense in birds in general, but here a different pathogenicity of both species is assumed. In cases of mycobacteriosis caused by M. genavense, gross pathological findings were described more often in psittacines than in passerines (20). This is in contrast to the findings presented here, given that in mycobacteriosis with isolation of M. genavense only, hepatosplenomegaly and the presence of granulomas were observed without significant differences in birds of both orders, but overall in a lower proportion of cases compared to M. avium infected birds. Hence, it is more likely that pathological findings depend on the causative mycobacterial species. This is further supported by the different histopathological presentation of lesions induced by these pathogens. Accumulations of epithelioid cell-like macrophages with numerous acid-fast rod-shaped bacteria in various organs were a characteristic finding in cases caused by M. genavense, resulting in hepatosplenomegaly without formation of granulomas and absence of gross necropsy findings (2, 18, 30). Organ distribution of mycobacteriosis was irrespective of the mycobacterial species and also of the bird order. However, the organs affected allow some conclusions on the route of infection. Manifestations of mycobacterial lesions in the small intestine point to oral ingestion, while manifestations in the lungs indicate airborne transmission of mycobacteria, which so far has only been suspected in birds (2, 9, 13, 18).

Reports of M. avium subsp. *hominissuis*-associated pathomorphological findings in pet birds or wild birds are rare, including one case from a blue fronted Amazon parrot (*Amazona aestiva*) with nontuberculous lesions (10), and one case of a Red-crested turaco (*Tauraco erythrolophus*) kept in a German zoo, which exhibited multiple granulomas within the body cavity and granulomas in various inner organs (32). Granulomas caused by M. avium subsp. *hominissuis* were described here in a red-crowned parakeet kept as individual pet bird, which revealed granulomatous lesions on the conjunctiva and the cervical air sacs. In contrast, mycobacteriosis without formation of granulomas caused by subsp. *hominissuis* was observed in a wild European robin. A mixed infection with both M. avium subspecies, here in a Tambourine dove, resulted in hepatosplenomegaly with granulomas, visceral dissemination of granulomas, as well as manifestation in the lung and small intestine. However, from 12 out of 20 water birds in a zoological garden naturally infected by subsp. *avium* and additionally infected by subsp. *hominissuis*, only three showed granulomatous lesions (9).

Mixed infections with M. avium and M. genavense or with different genotypes of M. avium subsp. *avium* have previously been reported in birds (9, 11, 16, 20, 29). Interestingly, one of the three birds with mycobacteriosis caused by a mixed infection in this study was a juvenile wild common kestrel, although mycobacteriosis in juvenile birds has been reported less frequently (2, 18, 30). However, there are no indications from the cases presented here and no reports that mixed infections result in more serious pathological lesions than infections with only one mycobacterial species or subspecies (9).

### Coinfections and other possible factors for immunosuppression.

Viral coinfections are common in cases of avian mycobacteriosis caused by M. genavense (17, 20, 21). This agrees with the results presented here and apparently also applies to M. avium subsp. *Avium*-induced mycobacteriosis in birds. Immunosuppression caused by a viral infection or by visceral coccidiosis in passerines seems to be a common trigger for infection with mycobacteria irrespective of the mycobacterial species. Despite this, infections with polyomavirus (33) and/or visceral coccids (34) are much more common than mycobacteriosis in passerine birds, so that other factors may play a role for the individual response to both mycobacterial species. Furthermore, mycobacterial lesions and transient colonization of the intestines at the same time were described in birds of the same species kept in the same aviary (9, 16). Environmental factors, like weather conditions and season, indoor housing with a lack of the sterilizing effects of UV irradiation, as well as individual stress related factors like breeding and molting, have been discussed as predisposing factors for the development of avian mycobacteriosis (35). The present data revealed no association between husbandry condition or gender of the birds and severity of mycobacteria-associated pathological lesions, causative mycobacterial species, or coinfections.

Interestingly, the single red-crowned parakeet housed indoors with isolation of M. avium subsp. *hominissuis* was without coinfections or comorbidities, which underlines that M. avium subsp. *hominissuis* is also pathogenic to birds.

### Comparison of genotyping methods.

The advantages of the genotyping methods used for M. avium isolates, MIRU-VNTR-analysis (based on variations in number of tandem repeats), and MLST (targeting sequence level variations in housekeeping genes), are the very good standardization of both methods and data analysis, and in particular the easy comparability of the results with other studies worldwide. Using results of both typing methods in combination, the discriminatory power of differentiation increased because results did not correlate. Band patterns of IS*901*-RFLP typing are also comparable between different studies, but this technique is time-consuming and needs a high amount of high-quality DNA from each isolate. While whole genome sequencing (WGS)-based SNP analysis for differentiation of isolates is much more sensitive, the procedure is demanding and has not yet been standardized. Currently, using WGS-based SNP analysis, it is possible to reveal transmission linkages in a known population (≤5 SNPs) (11) and to determine the diversity or similarity among isolates in a given study. However, accessibility of all raw reads is a prerequisite for comparison of SNPs of individual isolates from different studies.

Mycobacterium genavense is a very slow-growing mycobacterium that is difficult to cultivate (2, 31). Different kinds of media for subculture were tested also within the framework of this study, but until now without sufficient success to obtain enough DNA with the required quality for WGS. Therefore, only one method, MLST, was newly established here for M. genavense.

### Differentiation of isolates by genotyping.

Despite different regional origin and host species, the M. avium isolates showed a moderate genetic diversity using results of MIRU-VNTR and MLST typing: six combined genotypes for M. avium subsp. *avium* from 19 birds and three for subsp. *hominissuis* from three birds. It is well known and agrees with these results that obligate pathogenic subsp. *avium* genomes show lower diversity than opportunistic pathogenic subsp. *hominissuis* isolates (11, 22, 25, 26, 36). Furthermore, the total sequence identity of seven target genes determined by MLST in all M. genavense isolates confirms results of WGS-based SNP analysis, indicating a very low diversity among sequences of M. genavense isolates of different origin and much less genomic variation of M. genavense in comparison with M. avium subsp. *avium* (11).

The IS*901*-RFLP with BstEII and PvuII digestion was used here to subdifferentiate several M. avium subsp. *avium* isolates that share the same combined genotypes. However, IS*901*-RFLP revealed the same differentiation of isolates as the combination of MIRU-VNTR and MLST typing results. Using MIRU-VNTR typing only, the discriminatory power was lower than using IS*901*-RFLP typing, which also has been reported before (37).

Until now, this is the first study using a combination of MIRU-VNTR and MLST typing for differentiation of M. avium genomes and for differentiation of isolates from pet birds. Therefore, results can only be compared with publications that used one of these two methods. Altogether, 17 MIRU-VNTR M. avium subsp. *avium* genotypes have been published thus far (http://mac-inmv.tours.inra.fr); 11 of these types determined in birds or poultry (22, 37). Subspecies avium profiles INMV67 and INMV100 were most commonly found here as well as in another study from France where these genotypes were isolated not only from birds but also from poultry, cattle, pigs, a cat, and a wild boar (INMV67) and poultry, cattle, pigs, and a goat (INMV100), (22). In addition, genotype INMV67 was also isolated from AIDS patients in France (26). Furthermore, the M. avium subsp. *avium* reference strains ATCC 15769, ATCC 25291^T^, and ATCC 35712 isolated from hen or chicken represent this genotype (38). The genotype INMV90 isolated here from a wild juvenile European starling seems to be very rare and has been isolated twice before from cattle in France (22). These results illustrate differences in the distribution of these genotypes within various host species and in different regions of Europe, possibly based on their different phenotypical characteristics such as ability to survive and other virulence characteristics, or by chance. So far, the true causes for the different frequency of occurrence and spread are unknown. In our study, one new M. avium subsp. *avium* profile was suspected in the mixed isolate from Tambourine dove together with subsp. *hominissuis* profile INMV93, which was identified before in clinical isolates from 15 patients from Italy (39).

Mycobacterium avium subsp. *hominissuis* as a very common cause of nontuberculous mycobacterial infection in humans, pigs, and many other mammals was frequently differentiated by MIRU-VNTR analysis; to date about 117 profiles are deposited (http://mac-inmv.tours.inra.fr). So far, there are only a few M. avium subsp. *hominissuis* isolates originating from birds and, of these, even fewer that have been genotyped by MIRU-VNTR. The MIRU-VNTR profile new 1, detected here in a Red-crowned parakeet isolate, was identified before in two human isolates from Italy (39). Another profile, INMV51, was uniquely detected in an isolate of a Red-crested turaco kept in a German zoo (32) but was also determined in isolates from different mammals in Germany (Möbius, unpublished results) and in human, porcine, or bovine isolates in different European countries and Japan (22, 38–41). However, these MIRU-VNTR results give only some hints concerning the genetic similarity of the isolates, while differences in other genomic sequence regions are still possible as shown here using combined genotyping and in various studies worldwide (37, 38, 42), which may even be associated with phenotypic differences (39, 40).

The highly reliable molecular technique of MLST was developed to reveal genetic diversity across M. avium species as a whole and across the different subspecies based on sequence level variation in housekeeping genes (25, 36). Bird isolates in our study belonged to previously described sequence types (STs): to four STs of subsp. *avium* (ST21-23, ST40) and three STs of subsp. *hominissuis* (ST50, ST51, ST60). While ST21 was the most common genotype of bird isolates in this and another study (25), and ST22 was detected in several (this study) or only one bird isolates (25), Turenne et al. (36) found both STs only in one bovine and one porcine isolate, respectively. The ST23, in our study determined for one individual bird isolate, was identified in chicken, human, and a crane before (36). The subsp. *hominissuis* ST50, ST51, and ST60, here determined in one bird isolate each, have so far only been found before in human isolates (25). In the current study, the allele profile of the gene “*est*” could be completed for the ST50, ST51, and ST60 (25). However, the detection of these sequence types, also found in various other hosts, underscores the wide distribution of M. avium subsp. *avium* and subsp. *hominissuis* genotypes, as has already been demonstrated using MIRU-VNTR.

The mixed genotypes within single samples are caused either by multiple infections of individual birds or infections by multiple genotypes of the same mycobacterial species or subspecies. Definitions of mixed infections based on multiple genotypes or of within-host evolution vary, depending on the used typing method. Using WGS-based SNP analysis, distinct genotypes were separated by more than 12 SNPs (11). In the current study, we could identify mixed infections with both M. avium subspecies based on different and subspecies-specific MLST and MIRU-VNTR genotypes. Furthermore, three mixed isolates consisting of the two species M. genavense and M. avium were revealed by PCR. Allele diversity at one MIRU-VNTR locus only, as revealed in one M. avium subsp. *avium* clone, could arise from microevolution within the host following a single infection.

### Virulence, transmissions, and genotype.

In this study, an association of specific M. avium genotypes with distinct pathological findings could not be proven, contrary to results of other studies which used different methods and distinguished between virulent and nonvirulent M. avium genotypes (14, 43). However, studies using IS*901*-RFLP for strain characterization of subsp. *avium* concluded that individual genotypes are associated with the regional origin of animals (individual flocks), useful for revealing sources of infection but not for reliable virulence assessment (23). Here, too, a transmission of mycobacteria strains between birds of the same flock can be suspected in two cases: M. avium subsp. *avium* with combined genotype 5 (INMV100-ST22) among two pink headed fruit doves, and M. genavense between a green-backed twinspot and a black-rumped waxbill.

Differences in virulence of M. avium subsp. *avium* genotypes for birds can be assumed based on the presented results: a possible lower virulence of the combined genotype 4 and a possible higher virulence of the combined genotype 2. Such evidence will have to be verified in further studies with a larger number of affected birds or by infection studies.

### Summary.

In the present study, 50 cases of birds diagnosed with avian mycobacteriosis in routine diagnostics at the Clinic for Birds and Reptiles at the University Leipzig were examined for pathological findings, mycobacterial species causing mycobacteriosis, coinfections, and comorbidities. The cases included 45 pet birds, mainly psittacines and passerines, from different aviaries (household, in- and outdoor) and five wild birds from a total of 10 different federal states of Germany. Mycobacteria were successfully isolated from a total of 34 birds.

Avian mycobacteriosis in pet birds is equally caused by M. avium subsp. *avium* and M. genavense independent of bird order, gender, or husbandry conditions. Both species were also detected in wild birds. Some mixed or multiple infections were revealed. In individual cases M. avium subsp. *hominissuis* was identified as causative agent.

Granulomas were more often seen in mycobacteriosis caused by M. avium subsp. *avium* than in mycobacteriosis caused by M. genavense, and also in one case of mycobacteriosis caused by M. avium subsp. *hominissuis.* The characteristic pathomorphological findings seen in the small intestine and in the lung of about two thirds of the birds point to an oral as well as a pulmonal ingestion of mycobacteria.

Altogether, 80% of the birds examined here showed single or multiple coinfections with other pathogens independent of the mycobacterial isolate. Mycobacteriosis-related pathological findings were not associated with any coinfections.

The isolated M. avium subsp. *avium* showed moderate (subsp. *avium*) and high (subsp. *hominissuis*) diversity; altogether, nine combined genotypes were identified using MIRU-VNTR and MLST. An association between genotypes and pathological findings or bird orders could not be determined. Isolates of the genetically rather monomorphic species M. genavense could not be distinguished from each other by MLST.

Future studies will provide further knowledge about possible virulence differences of individual mycobacterial genotypes in birds and possible associations between mycobacterial species and susceptibility of specific bird orders.

## MATERIALS AND METHODS

### Cases included in the study.

Between 2017 and 2019, mycobacteriosis was diagnosed in 50 (4.7%) out of a total of 1,074 birds of different regional origin in Germany during routine necropsies at the Clinic for Birds and Reptiles, University Leipzig, including cytological, histopathological, parasitological, bacteriological, and mycological examinations as described elsewhere (44). These 50 birds included 35 species belonging to seven bird orders (Psittaciformes, Passeriformes, Columbiformes, Musophagiformes, Galliformes, Falconiformes, and Pelicaniformes). The classification of individual birds (order and species), age, gender, regional origin, bird husbandry, coinfections, and comorbidities, as well other information, are shown in Table 1.

Except a common kestrel (*Falco tinnunculus*) and a European starling (*Sturnus vulgaris*) which were juveniles, the birds were adult and of both genders (*n* = 27 males, *n* = 23 females). The birds originated from 10 different federal states of Germany. Two red-crowned parakeets (*Cyanoramphus novaezelandiae*), two blue-winged parrotlets (*Forpus xanthopterygius*), two estrildid finches (*Black-rumped waxbill [Estrilda troglodytes] and green-backed twinspot [Mandingoa nitidula]*), and three pink-headed fruit doves (*Ptilinopus porphyreus*) were from one bird keeper each; the other birds were kept in aviaries of different owners. Additionally, other birds with an origin-identical postal code were two red-crowned parakeets from North Rhine-Westphalia, one Fischer's lovebird (*Agapornis fischeri*), one yellow-collared lovebird (*Agapornis personatus*), and one common kestrel, as well as one European robin (*Erithacus rubecula*) and one green-backed twinspot from two different locations of Saxony. The Fischer's lovebird and the yellow-collared lovebird had been bought from a pet shop 6 months before death. All other birds had been housed in their individual environment for more than 6 months. The majority of birds were kept in pairs or groups, except two red-crowned parakeets, one yellow-crowned parakeet, one Fischer's lovebird, one Australian king parrot (*Alisterus scapularis*), and one Gouldian finch (*Chloebia gouldiae*) that were kept as single birds in the household. Beside the six single-kept birds, 16 birds were kept only in indoor aviaries in the household and the remaining 23 birds were kept in combined outdoor and indoor aviaries. The latter included one budgerigar, one swift parrot (*Lathamus discolour*), one white-throated magpie-jay (*Calocitta formosa*), and one Malagasy pond heron (*Ardeola idea*), which were kept in different zoos. Both European starlings, the common kestrel, the European robin, and one feral pigeon (*Columba livia* forma domestica) were wild birds.

Part of the psittacine birds (11 of 22 birds, 50%) and one Fischer's turaco (*Tauraco fischeri*) were presented at the clinic for diagnostics and treatment because they were emaciated. Four wild birds and one Fischer's lovebird were unable to fly and traumatic injuries with fractures of various bones were diagnosed. All birds died or were euthanized because of a poor prognosis. The remaining 33 birds were sent in dead for postmortem examination.

### Diagnosis of mycobacteriosis.

Impression smears from spleen, liver, lung, crop/oesophagus, proventriculus, and small and large intestine were stained with DiffQuik (Dade Behring, Marburg, Germany) during routine necropsy. In case of suspected granulomas, defined by proliferative white to yellow nodular foci in liver, spleen or lung, and/or in case of cytologically detected hyperplasia of macrophages, especially containing unstained bacterial rods, so called ghost-bacteria, a Ziehl-Neelsen staining was done and examined microscopically at ×1,000 magnification. Sections of skin, conjunctiva, bone marrow, visceral organs, and brain were fixed in 4.5% neutral buffered formalin for at least 24 h. Formalin-fixed samples were dehydrated, routinely embedded in paraffin wax, and sectioned at 4 μm. All sections were stained with hematoxylin and eosin (HE) and, in cases of suspected amyloidosis, with Congo red and periodic acid–Schiff (PAS) reaction. To verify acid-fast bacteria in the cytologically suspect samples, Ziehl-Neelsen stain and Fite-Faraco stain were used. The bird was considered mycobacteriosis positive when histopathological lesions consistent with mycobacteriosis and acid-fast bacteria were observed.

### Identification of coinfections and comorbidities.

In each case, DNA was isolated from 10 mg splenic tissue of all birds, from 15 mg liver tissue and 15 mg kidney tissue of psittacine and passerine birds by use of DNeasy blood and tissue kit (Qiagen, Hilden, Germany). Furthermore, RNA was isolated from 15 mg liver tissue of passerine birds, 15 mg brain tissue of passerine and psittacine birds, and 15 mg of neoplastic tissue in the chicken (Gallus gallus forma domestica) using RNeasy minikit (Qiagen).

Examination for apicomplexan parasites was performed in each bird using the DNA obtained from the spleen by amplifying the 18S rDNA and 28S rDNA as described previously (45). Amplification of mitochondrial cytochrome B gene was carried out for the detection of haemosporidian parasites in each bird using the DNA obtained from the spleen (46). In order to test for polyomaviruses and circoviruses in passerine and psittacine birds, DNA was isolated from kidney tissue in case of polyomaviruses, and liver tissue in case of circoviruses, and family-specific consensus nested-PCRs were performed as previously described (47, 48). RNA isolated from brain and liver was investigated for the presence of bornaviruses in psittacines and passerine birds, and Usutu virus in passerine birds using consensus RT-PCRs as described previously (49, 50). Furthermore, in the case of the chicken, RNA obtained from neoplastic tissue was used for detection of avian leucosis virus (51). Detailed information about PCRs used in this study is provided in Table S1.

### Isolation and culture of mycobacteria.

From most birds (46 of 50 birds), one acid-fast positive tested tissue was submitted frozen for isolation of mycobacteria. Various tissues were examined in case of one European starling, one European serin (*Serinus serinus*), one turquoise honeycreeper (*Dacnis cayana*), and one budgerigar (Table 1). The liver (*n* = 17) was the most commonly used organ, followed by the spleen (*n* = 14), small intestine (*n* = 12), lung (*n* = 8), granulomas from the serosa (*n* = 2), and joint and bone marrow (one each) (Table 1).

After defrosting, fat and connective tissue were removed from tissues. The intestine was opened and ingesta removed. Up to 1 *g* of sample was minced with scissors and transferred into a plastic bag containing 7 mL 0.9% hexadecyl pyridinium chloride. The samples were homogenized in a stomacher for 6 min, transferred to a 50-mL tube, and agitated on a shaker at 200 rpm for 10 min at room temperature (RT). Afterwards, they were incubated in upright position for 24 h at RT in the dark. After centrifugation at 1,880 × *g* for 20 min at RT, supernatants were discarded, and the pellet resuspended with 1.2 mL of sterile phosphate-buffered saline (pH 7.2). Then, 150 μL of the pellet were transferred into each of three tubes of Middlebrook 7H9 broth (Becton, Dickinson, Heidelberg, Germany) supplemented with 10% Middlebrook Oleic acid, Albumin, Dextrose, and Catalase (OADC) enrichment (Becton, Dickinson), 2 mg/L Mycobactin J (IDvet, Grabels, France), and 10% PANTA (Polymyxin B, Amphotericin B, Nalidixic acid, Trimethoprim, Azlocillin, all Sigma-Aldrich, Taufkirchen, Germany), and on one slant each of Loewenstein-Jensen medium with glycerol and PACT (Polymexin B, Amphotericin B, Carbenicillin, Trimethoprimlactat), Coletsos medium with pyruvate, glycerol, and PACT (both Artelt Enclit, Borna, Germany), and Herrold’s egg yolk medium with Mycobactin J and ANV (Amphotericin, Nalidixic acid, Vancomycin) (Becton, Dickinson). The solid media and one tube of Middlebrook 7H9 broth were incubated at 37°C for 8 and 12 weeks, respectively, and the remaining two tubes of Middlebrook 7H9 broth were incubated at 42°C for 12 weeks. For genotyping, M. avium isolates were propagated in Middlebrook 7H9 broth and subcultivated on Coletsos medium and Loewenstein-Jensen medium and incubated at 37°C.

### Mycobacterial species, subspecies, and variant identification by PCR.

DNA was extracted from 100 μL of broth culture, or by suspending a loopful of bacterial colony material in 100 μL distilled water, heating for 20 min at 80°C, ultrasonication (35 Hz) for 10 min, heating for 10 min at 100°C, and centrifugation for 5 min at 12,000 rpm (15,300 × *g*). The supernatant was transferred to a new tube and again centrifuged for 5 min at 12,000 rpm. The supernatant contained the DNA. DNA concentration was measured using a NanoDrop spectrophotometer.

For species, subspecies, and variant identification, different PCR analyses were applied, targeting Mycobacterium genus-specific 16S rRNA gene (52), M. genavense specific 21 kDa protein gene (53), and M. avium subspecies-specific insertions sequences (IS) including IS*1245* and IS*901.* The confirmed M. avium isolates were identified to be M. avium subsp. *avium*, as they were positive for IS*1245* (54) and positive for variant specific IS*901* (4). In contrast, M. avium subsp. *hominissuis* isolates were negative for IS*901*, but positive for IS*1245*, and positive for the flanking region of IS*901* FR300 without the IS*901* element (4, 55). Mixed infections with both M. avium subspecies were revealed using the mycobacterial interspersed repetitive-unit-variable-number tandem-repeat (MIRU-VNTR) analysis results.

### Molecular differentiation (genotyping).

All M. avium isolates were characterized by two methodologies: MIRU-VNTR analysis (26) and multilocus sequence typing (MLST) (25, 36). Selected M. avium subsp. *avium* isolates were additionally genotyped by restriction fragment length polymorphism (RFLP) analysis based on the insertion sequence IS*901* (IS*901*-RFLP) (7).

All M. genavense isolates grew only on liquid medium. Subcultivation was not successful on solid or in liquid media. Therefore, the amount of available bacterial material was only sufficient for standard DNA isolation (see above), species identification, and establishment of one new method for genotyping. M. genavense isolates were characterized by MLST analysis newly established for this species in this study.

For MIRU-VNTR analysis, differences in the number of tandem repeat sequences (=different alleles) were detected by PCRs targeting specific loci of the M. avium genome: MIRU-VNTR Loci 292, X3, 25, 47, 3, 7, 10, and 32 (26). The PCR conditions used were described previously (56), (for the updated annealing temperatures, see Table S1) and tandem repeat numbers were determined as shown in the supplemented material of Radomski et al. (2010) (22). Results were arranged according to the mentioned order of loci and, based on the INMV classification database (http://mac-inmv.tours.inra.fr/), the so-called INMV profiles were determined. M. avium isolates show variant-specific MIRU-VNTR profiles, previously designated as subspecies- or lineage-specific profiles. Unknown profiles in mixed isolates were assigned by cluster analysis.

MLST analysis exploits variations in the DNA sequence of several conserved genes, and reveals specific sequence profiles for each strain. In brief, target genes for M. avium isolates comprise the loci in *recF*, *gnd1*, *lipT*, *pepB*, and a putative esterase, designated as “*est*” (25). After amplification of a region of approximately 1 kb, the PCR product was purified using the QIAquick PCR purification kit (Qiagen, Hilden, Germany) and sequenced by the sequencing service at Eurofins Genomics GmbH (Germany). Resulting sequences were compared with the corresponding sequences of reference strain M. avium 104 (NC_008595.1). The detection of SNPs at specific variable positions in the respective target loci was used to assign specific sequence alleles. The specific allele distributions based on the results for the individual different target genes are concatenated to individual allelic profiles and defined as the respective ST of each strain (25, 36). All determined sequence alleles and ST were assigned accordingly. MLST genotypes of mixed M. avium isolates revealed by MIRU-VNTR were checked and identified manually using the original sequence chromatograms and analyzing the mixed peaks at the above-mentioned distinct variable positions and the control of lineage (variant) specific SNPs.

For the establishment of a novel MLST method for M. genavense, nine out of 10 conserved enzyme encoding genes selected for MLST in M. avium (25, 36) were identified in M. genavense by comparison of sequences and annotations between M. avium strain 104 (NC_008595.1) and M. genavense strain ATCC 51234 (NZ_JAGZ01000001), except the putative esterase “*est*.” The DNA sequence identity for eight genes was ≤ 88% (in M. avium 104 versus M. genavense) suggesting sufficient genetic variability for differentiation. For seven genes (*recF*, *gnd1*, *lipT*, *pepB*, *sodA*, *aspB*, and *groL1*) primers were designed using Geneious prime (version 2021.0.1). Genes, primers, and regions of analysis in M. genavense are presented in Table S2. The amplification of target regions in these genes was performed in a 50 μL final reaction mixture volume consisting of approximately 150 ng of DNA, 1x PCR buffer (Qiagen), 2.5 mM MgCl_2_, 5% DMSO, 0.2 mM each deoxynucleotide triphosphates (dNTP mix, Qiagen), 0.6 μM each primer, and 1 U of HotStarTaq DNA polymerase (Qiagen). PCR was performed with an Eppendorf Mastercycler nexus GX2 using the following conditions: 95°C for 15 min; 38 cycles with intervals of 96°C (15 s), 55°C (1 min), and 72°C (1 min); 72°C for 10 min; and holding at 4°C. PCR product (5 μL) was visualized on a 1% agarose gel containing ethidium bromide. The remaining PCR product was purified as described before for M. avium subsp. avium MLST and submitted for sequencing using forward and reverse primers. For all isolates, sequencing results from both directions were analyzed in Geneious using “.ab” files, including the DNA sequence, quality information, and the chromatogram. Single nucleotide variants (SNVs) identified in some isolates only in one sequence direction (mostly characterized by a low quality of base calls) were not accepted.

For IS*901*-RFLP analysis of M. avium isolates, genomic DNA was prepared by the cetyltrimethylammonium bromide method (57). For IS*901*-RFLP, two digestion enzymes were used: BstEII and PvuII. Two individual band patterns per isolate documented on images were compared and designated according to the known IS*901*-RFLP (PvuII) type designation (43, 58) or new designations.

### Statistical analysis.

Statistical analysis was performed using SPSS 27 (IBMSPSS Headquarters, Chicago, Illinois). All data were binary categorized. *Chi*-squared statistics using the Fisher’s exact test and the *phi* coefficient (*φ*) for measuring associations between the variables were performed. Using Fisher’s exact test, *P ≤ *0.05 was considered significant association. Binary logistic regression analysis was used with each of both mycobacterial species for the following criteria: birds kept only indoors, husbandry, gender, emaciation, granulomas, hepatosplenomegaly, affected organs (viscerally disseminated, small intestine, lung), without gross pathological findings, coinfections with endoparasites, coinfections with bacteria, without coinfections, trauma, passerines, and psittacines. The contribution of each criterion was evaluated by the likelihood-ratio test, and determining the odd ratios (*OR*) with two independent variables was supported by a significant *P* value. Furthermore, binary logistic regression analysis was used for each mycobacterial species and polyomavirus or *Macrorhabdus ornithogaster* in psittacines and passerines, polyomavirus or *M. ornithogaster* with husbandry, body condition, granulomas, hepatosplenomegaly, affected organs, coinfections with endoparasites, coinfection with bacteria, without coinfections, without gross pathological findings in passerines and psittacines, and only in passerines, each mycobacterial species with visceral coccids and endoparasites or visceral coccids with husbandry, body condition, granulomas, hepatosplenomegaly, affected organs, coinfections with polyomavirus, bacteria, without coinfections, without gross pathological findings, and trauma. Additionally, binary logistic regression analysis of passerines or psittacines with all mentioned criteria, except visceral coccids, was tested. The significance level for all criteria was *P ≤ *0.05.

The number of birds with identical M. avium genotypes was too small for statistical analyses of possible association between genotypes and individual pathological findings, bird order, bird husbandry, or regional origin.
