# Supplementary material for: Mycobacteriosis in Various Pet and Wild Birds from Germany: Pathological Findings, Coinfections, and Characterization of Causative Mycobacteria
Source: Microbiol Spectr. 2022 Jul 19;10(4):e00452-22. doi: 10.1128/spectrum.00452-22 (PMC9430480; doi:10.1128/spectrum.00452-22)
Supplement: Supplemental file 1 — Supplemental material. Download spectrum.00452-22-s0001.pdf, PDF file, 0.4 MB [file spectrum.00452-22-s0001.pdf]

Supplemental Material

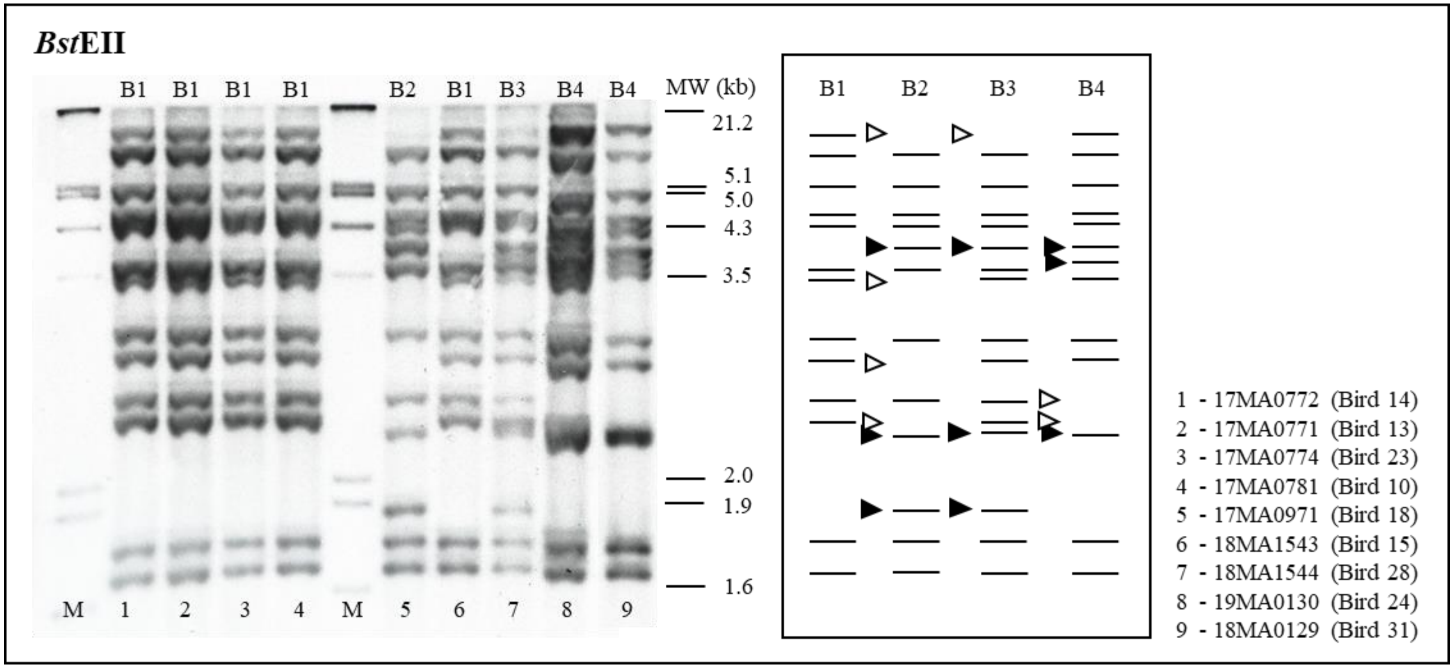

Figure S1. IS*901*-RFLP profiles of *Bst*EII-digested genomic DNA from nine selected *M. avium* subsp. *avium* strains. The letters above the lines designate IS*901*-RFLP (*Bst*EII) types, new in this study. Schematic figure to the right represents the type designation of different band pattern. Lanes M represent the molecular weight marker III (Roche Diagnostics).

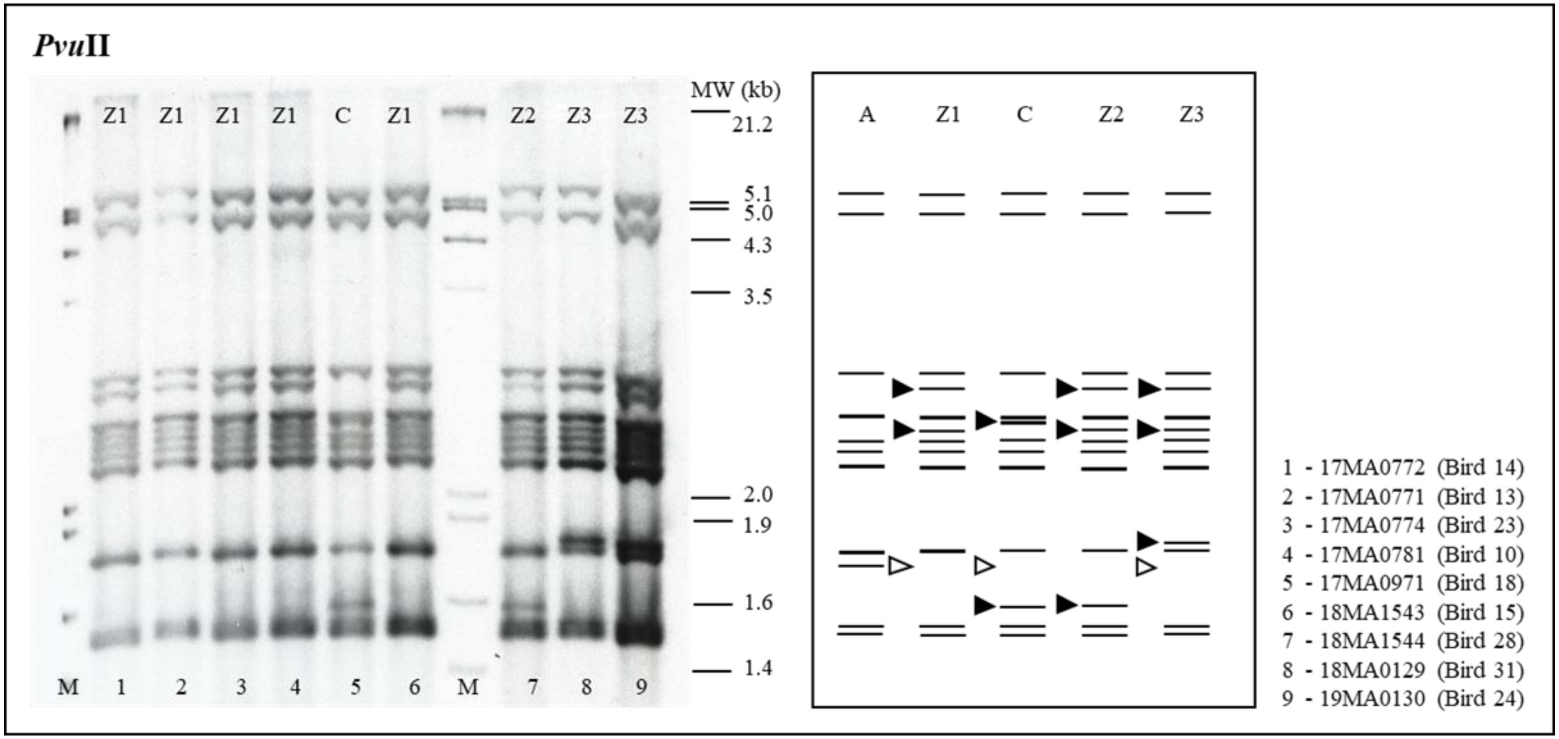

Figure S2. IS*901*-RFLP profiles of *Pvu*II-digested genomic DNA from nine selected *M. avium* subsp. *avium* strains. The letters above the lines designate the identified IS*901*-RFLP types. Schematic figure to the right represents the designation of distinct band pattern and includes IS*901*-RFLP type A as reference according to (1) and (2). Types Z1, Z2, and Z3 show new profiles, type C was published as stated before. Lanes M represent the molecular weight marker III (Roche Diagnostics).

Table S1. Details of PCR reactions used in this study

Examination target	PCR method	Material	PCR-target Region	Forward-Primer (5' - 3')<br>Reverse-Primer (5' - 3')	Annealing Temp.	Ref.
**1. Parasites**						
Apicomplexan parasites	conventional PCR	rRNA	18S rRNA	CCC ATG CAT GTC TAA GTA TAA GC<br>CAC TGC CAC GGT AGT CCA ATA C	58°C	(3)
	conventional PCR	rRNA	28S rRNA	TAC CCG CTG AAC TTA AGC<br>CCA CCA AGA TCT GCA CTA G	58°C	(3)
Haemosporidian parasites	conventional PCR	mtDNA	mitochondrial Cytochr. B gene	CAT ATA TTA AGA GAA ITA TGG AG<br>ATA GAA AGA TAA GAA ATA CCA TTC	58°C	(4)
Plasmodidae/Haemoproteidae	nested PCR 1			ATG GTG CTT TCG ATA TAT GCA TG<br>GCA TTA TCT GGA TGT GAT AAT GGT	58°C	
Leucocytozoon	nested PCR 2			ATG GTG TTT TAG ATA CTT ACA TT<br>CAT TAT CTG GAT GAG ATA ATG GIG C	58°C	
**2. Viruses**						
Polyomavirus	consensus nested PCR	DNA	Virus protein 1	CCA GAC CCA ACT ARR AAT GAR AA<br>AAC AAG AGA CAC AAA TNT TTC CNC C	46°C	(5)
				ATG AAA ATG GGG TTG GCC CNC TNT GYA ARG<br>CCC TCA TAA ACC CGA ACY TCY TCH ACY TG	56°C	
Circovirus	consensus nested PCR	DNA	Re-encoding region	AGA GGT GGG TCT TCA CNH TBA AYA A<br>AAG GCA GCC ACC CRT ARA ART CRT C	46°C	(6)
				AGC AAG GAA CCC CTC AYY TBC ARG G<br>ACG ATG ACT TCN GTC TTS MAR TCA CG	56°C	
Bornavirus	consensus RT-PCR	RNA	Nucleocapsid gene	CCH CAT GAG GCT ATW GAT TGG ATT AAC G<br>GCM CGG TAG CCN GCC ATT GTD GG	48.5°C	(7)
		RNA	Matrix gene	GGR CAA GGT AAT YGT YCC TGG ATG GCC<br>CCA ACA CCA ATG TTC CGA AGM CG	48.5°C	
Usutu virus	consensus RT-PCR	RNA	Nonstructural protein 1 gene	CGT TCT CGA CTT TGA CTA<br>GCT AGT AGT AGT TCT TAT GGA	58°C	(8)
Avian leucosis virus	RT-PCR	RNA	polymerase gene	GGA TGA GGT GAC TAA GAA AG<br>GGG AGG TGG CTG ACT GTG T	48°C	(9)

Examination target	PCR method	Material	PCR-target Region	Forward-Primer (5' - 3')<br>Reverse-Primer (5' - 3')	Annealing Temp.	Ref.
**3. Mycobacterial species and subspecies**						
Mycobacterium (*M.*) genus	conventional PCR	gDNA	16SrRNA gene	GAG AGT TTG ATC CTG GCT CAG<br>TGC ACA CAG GCC ACA AGG GA	60°C	(10)
M. genavense	conventional PCR	gDNA	21 kDa protein	TGC ACG GCA ACA TCA AAG AC<br>CGC CGG AAT CTG CAT CAC	62°C	(11)
M. avium	conventional PCR	gDNA	IS*1245*	GCC GCC GAA ACG ATC TAC<br>AGG TGG CGT CGA GGA AGA C	66°C	(12)
M. avium subsp. *avium*	conventional PCR	gDNA	IS*901*	GCA ACG GTT GTT GCT TGA AA<br>TGA TAC GGC CGG AAT CGC GT	60°C	(13)
M. avium, <u>not</u> subsp. *avium*	conventional PCR	gDNA	FR300*	CAG CCA GCC GAA TGT CAT CC<br>CAA CTC GCG ACA CGT TCA CC	65°C	(13, 14)
**4. MIRU-VNTR genotyping of *M. avium*)**						
	conventional PCR	gDNA	Locus 292	CTT GAG CAG CTC GTA AAG CGT<br>GCT GTA TGA GGA AGT CTA TTC ATG G	58°C	(15-17)
	conventional PCR	gDNA	Locus X3	AAC GAG AGG AAG AAC TAA GCC G<br>TTA CGG AGC AGG AAG GCC AGC GGG	62°C	(15-17)
	conventional PCR	gDNA	Locus 25	GTC AAG GGA TCG GCG AGG<br>TGG ACT TGA GCA CGG TCA T	55°C	(15-17)
	conventional PCR	gDNA	Locus 47	CGT TGC GAT TTC TGC GTA GC<br>GGT GAT GGT CGT GGT CAT CC	64°C	(15-17)
	conventional PCR	gDNA	Locus 3	CAT ATC TGG CAT GGC TCC AG<br>ATC GTG TTG ACC CCA AAG AAA T	60°C	(15-17)
	conventional PCR	gDNA	Locus 7	GAC AAC GAA ACC TAC CTC GTC<br>GTG AGC TGG CGG CCT AAC	60°C	(15-17)
	conventional PCR	gDNA	Locus 10	GAC GAG CAG CTG TCC GAG<br>GAG AGC GTG GCC ATC GAG	54°C	(15, 16)
	conventional PCR	gDNA	Locus 32	CCA CAG GGT TTT TGG TGA AG<br>GGA AAT CCA ACA GCA AGG AC	57°C	(15-17)

FR300* - IS*901*-flanking region only (without IS*901*) in *M. avium* subsp. *hominissuis*

Table S2. MLST for *M. genavense* (Mg): genes, primers and regions of analysis (this study)

**Gene**	**Gene name**	**Gene Size (bp)**	**Genome Locus_taq in JAGZ01 (Mg)**	**Scaffold**	**Primer sequence, 5' - 3'**	**Primer name**	**Size (bp)**	**Position on gene**	**MLST analysis:**
recF	DNA replication/ repair protein RecF	1164	T428_RS0119940	10	ATTTTCGGTCCTGGGCACAA<br>GGACATCTTCCAGTACCGCC	MG_recF_F<br>MG_recF_R	1,056	29 to 1084	100-1030 (930 bp)
gnd1	phosphogluconate dehydrogenase	1458	T428_RS28255	6	CGATCTCCAACCTGAGAGGC<br>CTCGTCGAAGGCCTCTTTGA	MG_gnd1_F<br>MG_gnd1_R	1,190	-26 to 1163	60-1080 (1020 bp)
lipT	carboxylesterase	1502	T428_RS26610	3	ACGGATTCACCAACTGTGCT<br>GGTGGGCAACATCTTCAGGA	MG_lipT_F<br>MG_lipT_R	820	170 to 989	225-950 (725 bp)
pepB	leucryl amino-peptidase	1548	T428_RS0101645	1	GAATTCCGCAGCGACAAGAC<br>CTCGCAGATGTCTTCCAGCA	MG_pepB_F<br>MGpepB_R	1,086	454 to 1539	510-1480 (970 bp)
sodA	superoxide dismutase	624	T428_RS0119670	10	TGATTACGCTGAGCCTGTCG<br>TCAGCCGAAAATCAAGCCCT	MG_sodA_F<br>MG_sodA_R	698	-74 to 624	1-563 (563 bp)
aspB	aminotransferase	1107	T428_RS0112880	5	GATCTGGTCAACCTCTCGGC<br>CGGCCAACAACTTCGAACAG	MG_aspB_F<br>MG_aspB_R	925	46 to 970	98-925 (827 bp)
groL1	Chaperonin GroEL	1623	T428_RS0117645	8	ACCCAACAACAAGTCGACGT<br>GATCGTGTCGTCCTTGCTGA	MG_groL_F<br>MG_groL_R	1,031	-44 to 987	12-930 (918 bp)

Table S3. Multilocus sequence alignments of sequence types (STs) identified among 20 German avian *M. avium* strains. All strains were examined in five target genes (*recF*, *gnd1*, *lipT*, *pepB*, and "*est*"). Alleles were designated and STs were identified based on the numbering system published (18, 19). *M. avium* 104 (*M. avium* subsp. *hominissuis*) was used as reference strain with ST1. Identity of nucleotides with ST1 is indicated with a dot. Variable positions are represented by SNPs.

target gene	*recF*		*gnd*		*lipT*	
nucleotide	111 (positions 31–33)		111 (positions 17–19)			
position	222233444555567777777778889999000		1123444567889999000		1122233445566666677888	
(top to	000129045045630136778890661369012		6846047523190557003		3548914025803355929346	
down)	124040273432767516492768055773543		5476581272699455898		8968355568232947732144	
ST		Allele		Allele		Allele
1	GACGACCGCGCCGCCGCGGCGTCGAGTTCGCAC	1	AGΔCCCGCTGGCCACTTTG	1	TCAGCGCTTCTCCCTACTCTTG	1
21	.GG.G.T....TAG.T..A..GAAGC...CG.G	6	..-.G..............	7	...................CC.	4
22	.GG.G.T....TAG.T..A..GAAGC...CG.G	6	..-.G..............	7	...................CC.	4
23	.GG.G.T....TAG.T..A..GAAG....CG.G	7	..-.G..........C...	7	....T..............CC.	5
40	.GG.G.T.....AG.T..A..GAAG....CG.G	8	..-.G..............	7	...................CC.	4
50	.G..GTT.TTA..G.....A.G.A...C.C.GG	5	T...G.CTCAA..G.CGC.	3	....	1
51	.GG.G.T......................C...	13	.A..G.......TG.C..A	10	....	1
60	.G..GTT.TTA..G.....A.G.A...C.C.GG	5	T...G.CTCAA..G.CGC.	3	CAG..C.C..C..G...C.C..	3

target gene	*pepB*		*est*	
nucleotide	111111 (positions 12–17)			
position	56666888899012222		111122334444555555567778 (positions 5–28)	
(top to	90028124955300345		5558233899452567116788820040	
down)	77932980937519924		4595628326716159474925871871	
ST		Allele		Allele
1	CACAAAATCGGCCCGCG	1	CCTACCGCGCGCCTGACGGCGGAGCCGA	1
21	..T.C.GC...T.G..C	5	GG..TT....AT.G........GC....	3
22	..T.C.GC...T.G..C	5	GG..TT....ATTG........GC....	10
23	..T.C.GC...T.G..C	5	GG..TT....AT.G........GC....	3
40	..T...GC...T.G..C	5	GG..TT....AT.G........GC....	3
50	....	1	.G.GTT..........TA.AACGC....	11
51	..TCCGG..A......C	8	....	1
60	....	1	.G.GTT..........TA.AACGC....	11

References

1. Ritacco V, Kremer K, van der Laan T, Pijnenburg JE, de Haas PE, van Soolingen D. 1998. Use of IS901 and IS1245 in RFLP typing of Mycobacterium avium complex: relatedness among serovar reference strains, human and animal isolates. Int J Tuberc Lung Dis 2:242-51.
2. Dvorska L, Bull TJ, Bartos M, Matlova L, Svastova P, Weston RT, Kintr J, Parmova I, Van Soolingen D, Pavlik I. 2003. A standardised restriction fragment length polymorphism (RFLP) method for typing Mycobacterium avium isolates links IS901 with virulence for birds. J Microbiol Methods 55:11-27.
3. Schmidt V, Dyachenko V, Aupperle H, Pees M, Krautwald-Junghanns ME, Daugschies A. 2008. Case report of systemic coccidiosis in a radiated tortoise (Geochelone radiata). Parasitology Research 102:431-436.
4. Hellgren O, Waldenstrom J, Bensch S. 2004. A new PCR assay for simultaneous studies of Leucocytozoon, Plasmodium, and Haemoproteus from avian blood. Journal of Parasitology 90:797-802.
5. Johne R, Enderlein D, Nieper H, Muller H. 2005. Novel polyomavirus detected in the feces of a chimpanzee by nested broad-spectrum PCR. J Virol 79:3883-7.
6. Halami MY, Nieper H, Muller H, Johne R. 2008. Detection of a novel circovirus in mute swans (Cygnus olor) by using nested broad-spectrum PCR. Virus Research 132:208-212.
7. Kistler AL, Gancz A, Clubb S, Skewes-Cox P, Fischer K, Sorber K, Chiu CY, Lublin A, Mechani S, Farnoushi Y, Greninger A, Wen CC, Karlene SB, Ganem D, DeRisi JL. 2008. Recovery of divergent avian bornaviruses from cases of proventricular dilatation disease: Identification of a candidate etiologic agent. Virology Journal 5.
8. Jost H, Bialonski A, Maus D, Sambri V, Eiden M, Groschup MH, Gunther S, Becker N, Schmidt-Chanasit J. 2011. Short Report: Isolation of Usutu Virus in Germany. American Journal of Tropical Medicine and Hygiene 85:551-553.
9. Smith LM, Brown SR, Howes K, McLeod S, Arshad SS, Barron GS, Venugopal K, McKay JC, Payne LN. 1998. Development and application of polymerase chain reaction (PCR) tests for the detection of subgroup J avian leukosis virus. Virus Research 54:87-98.
10. Kirschner P, Rosenau J, Springer B, Teschner K, Feldmann K, Bottger EC. 1996. Diagnosis of mycobacterial infections by nucleic acid amplification: 18-month prospective study. Journal of Clinical Microbiology 34:304-312.
11. Chevrier D, Oprisan G, Maresca A, Matsiota-Bernard P, Guesdon JL. 1999. Isolation of a specific DNA fragment and development of a PCR-based method for the detection of Mycobacterium genavense. Fems Immunology and Medical Microbiology 23:243-252.
12. Guerrero C, Bernasconi C, Burki D, Bodmer T, Telenti A. 1995. A novel insertion element from Mycobacterium avium, IS1245, is a specific target for analysis of strain relatedness. J Clin Microbiol 33:304-7.
13. Kunze ZM, Portaels F, McFadden JJ. 1992. Biologically distinct subtypes of Mycobacterium avium differ in possession of insertion sequence IS901. J Clin Microbiol 30:2366-72.
14. Bartos M, Hlozek P, Svastova P, Dvorska L, Bull T, Matlova L, Parmova I, Kuhn I, Stubbs J, Moravkova M, Kintr J, Beran V, Melicharek I, Ocepek M, Pavlik I. 2006. Identification of members of Mycobacterium avium species by Accu-Probes, serotyping, and single IS900, IS901, IS1245 and IS901-flanking region PCR with internal standards. J Microbiol Methods 64:333-45.

15. Radomski N, Thibault VC, Karoui C, de Cruz K, Cochard T, Gutierrez C, Supply P, Biet F, Boschiroli ML. 2010. Determination of genotypic diversity of Mycobacterium avium subspecies from human and animal origins by mycobacterial interspersed repetitive-unit-variable-number tandem-repeat and IS1311 restriction fragment length polymorphism typing methods. J Clin Microbiol 48:1026-34.
16. Thibault VC, Grayon M, Boschiroli ML, Hubbans C, Overduin P, Stevenson K, Gutierrez MC, Supply P, Biet F. 2007. New variable-number tandem-repeat markers for typing Mycobacterium avium subsp. paratuberculosis and M. avium strains: comparison with IS900 and IS1245 restriction fragment length polymorphism typing. J Clin Microbiol 45:2404-10.
17. Mobius P, Luyven G, Hotzel H, Kohler H. 2008. High genetic diversity among Mycobacterium avium subsp. paratuberculosis strains from German cattle herds shown by combination of IS900 restriction fragment length polymorphism analysis and mycobacterial interspersed repetitive unit-variable-number tandem-repeat typing. J Clin Microbiol 46:972-81.
18. Kolb J, Hillemann D, Mobius P, Reetz J, Lahiri A, Lewin A, Rusch-Gerdes S, Richter E. 2014. Genetic characterization of German Mycobacterium avium strains isolated from different hosts and specimens by multilocus sequence typing. Int J Med Microbiol 304:941-8.
19. Turenne CY, Collins DM, Alexander DC, Behr MA. 2008. Mycobacterium avium subsp. paratuberculosis and M. avium subsp. avium are independently evolved pathogenic clones of a much broader group of M. avium organisms. J Bacteriol 190:2479-87.
